# Complete chloroplast genome sequence and phylogenetic analysis of dragon fruit (*Selenicereus undatus* (Haw.) D.R.Hunt)

**DOI:** 10.1080/23802359.2021.1903356

**Published:** 2021-03-24

**Authors:** Jin Liu, Zi-yan Liu, Cheng Zheng, Ying-feng Niu

**Affiliations:** Yunnan Institute of Tropical Crops, Xishuangbanna, China

**Keywords:** *Selenicereus undatus* (Haw.) D.R.Hunt, Chloroplast genome sequence

## Abstract

Selenicereus undatus (Haw.) D.R.Hunt is a member of the family Cactaceae. The chloroplast genome of S. undatus was sequenced, assembled, and annotated in the present study. The chloroplast genome was 133,326 bp in length, consisting of a typical quadripartite circle: a large single-copy region of 68,256 bp, two inverted repeat regions of 21,677 bp, and a small single copy region of 21,716 bp. A total of 120 predicted genes were identified, and a maximum likelihood was constructed, placing S. undatus as the sister taxon of Lophocereus schottii and Carnegiea gigantea, other members of the family Cactaceae.

*Selenicereus undatus* (Haw.) D.R.Hunt 1918, which was previously known as *Hylocereus undatus* before the genus *Hylocereus* was determined to be deeply embedded within the genus *Selenicereus* (Korotkova et al. 2017), also called dragon fruit, pitaya, or strawberry pear, is a member of the family Cactaceae native to tropical areas of North, Central, and South America (Barbeu 1990; Zhuang et al. 2012). It is now being cultivated throughout the world, especially in Asian countries such as Vietnam, Taiwan, Malaysia, and the Philippines (Mizrahi et al. 1997). Several studies have shown that *S. undatus* fruits are a good source of minerals, glucose, fructose, dietary fiber, and vitamins (Barbeu 1990; Wu and Chen 1997), and a natural colorant from the peel of pitaya fruit can be used in a wide range of foods (Rodrigues et al. 1995). In this study, the chloroplast genome of *S. undatus* was reported.

Fresh samples of *S. undatus* were collected from Xishuangbanna Tropical Flowers and Plants Garden (22.015407 N, 100.789477 E), Yunnan, China and frozen in liquid nitrogen. The specimen was deposited at the herbarium of the Yunnan Institute of Tropical Crops (http://www.yitc.com.cn/, fzzx@yitc.com.cn) under the voucher number of YITC-2020-FZ-C-022. Its genomic DNA was extracted using the DNeasy Plant Mini Kit (Qiagen. Hilden, Germany), and DNA quality was characterized using the Nano-Drop 2000 spectrometer (Thermo Fisher Scientific, Waltham, MA, USA). The DNA library was constructed with insert sizes of 350 bp, and paired-end (PE) sequencing was conducted on the Illumina HiSeq 2500 platform (Illumina, San Diego, CA, USA). Approximately 7.5 Gb of raw data were thus obtained and then assembled using GetOrganelle (Jin et al. 2020) and cross-validated by SPAdes v. 3.5.0 (http://soap.genomics.org.cn/soapdenovo.html). The assembled genome was annotated using GeSeq (Tillich et al. 2017) and CpGAVAS2 (Shi et al. 2019), followed by manual examination using Geneious 11.1.5 software (Kearse et al. 2012), and the sequence of genes with uncertain annotation results was verified by cDNA sequencing. Then, the chloroplast genome sequence was submitted to GenBank (accession number, MT884001).

The *S. undatus* chloroplast genome was 133,326 bp in length and relatively conservative in its structure relative to those of most other plant species (Liu et al. 2018). The genome consisted of a typical quadripartite circle consisting of the following regions: a large single-copy region (LSC, 68,256 bp), two inverted repeat regions (IRs, 21,677 bp), and a small single-copy region (SSC, 21,716 bp). The GC content of the LSC, IRs, and SSC regions and the whole chloroplast genome was 36.26%, 34.98%, 39.69%, and 36.40%, respectively. Across the whole chloroplast genome, the A, T, G, and C bases numbered 42,093, 42,699, 23,992, and 24,542, respectively. A total of 120 genes were predicted, including 76 protein-coding genes, 4 pseudogenes, 36 tRNA genes, and 4 rRNA genes. The protein-coding genes are involved in photosystem I, photosystem II, the cytochrome b/f complex, ATP synthase, NADH dehydrogenase, RNA polymerase, and other biological functions. Four protein-coding genes, including *atpF* and *rps16*, contained one intron, while three protein-coding genes, including *ycf3* and *rps12*, contained two introns.

The sequencing and assembly of chloroplast genomes of several cactaceous species have been completed (Sanderson et al. 2015; Majure et al. 2019; Solórzano et al. 2019; Kohler et al. 2020), including *Carnegiea gigantea* (113,064 bp), *Cylindropuntia bigelovii* (125,158 bp), *Opuntia quimilo* (150,374 bp), *Mammillaria albiflora* (110,789 bp), *Mammillaria albiflora* (108,561 bp), *Mammillaria crucigera* (115,505 bp), *Mammillaria huitzilopochtli* (115,886 bp), *Mammillaria solisioides* (115,356 bp), *Mammillaria supertexta* (116,175 bp), and *Mammillaria zephyranthoides* (107,343 bp). Compared with these species, the chloroplast genome of *S. undatus* is only smaller than that of *Opuntia quimilo*, which has a very long LSC region (104,475 bp) accounting for 67.48% of its entire chloroplast genome. In addition, the chloroplast genome structure and the number of genes of most cactaceous species also differ substantially.

To investigate the phylogenetic relationship of *S. undatus* to other species in the order Caryophyllales, a maximum likelihood tree based on the DNA sequences of the protein-coding genes common to chloroplast genomes of all species was constructed. The 24 species used for phylogenetic tree construction belong to six different families within the order Caryophyllales. Among them, 11, 3, 5, 2, 2, and 1 species belonged to the families Cactaceae, Portulacaceae, Montiaceae, Aizoaceae, Basellaceae, and Talinaceae, respectively, the last of which consisted of the single species *Talinum paniculatum*, which was used as the outgroup. The 24 complete chloroplast genomes of the order Caryophyllales were aligned using MAFFT (Katoh and Standley 2013), and maximum likelihood analysis was performed using RAxML based on the GTRGAMMA substitution model (Stamatakis 2014) with 1000 bootstrap replicates, As shown in Figure 1, *S. undatus* is posited as the sister taxon to *Lophocereus schottii* and *Carnegiea gigantea*, all of which belong to the family Cactaceae. This study provides a foundation for future studies on the genetic diversity and phylogenetics of the order Caryophyllales.

**Figure 1.**
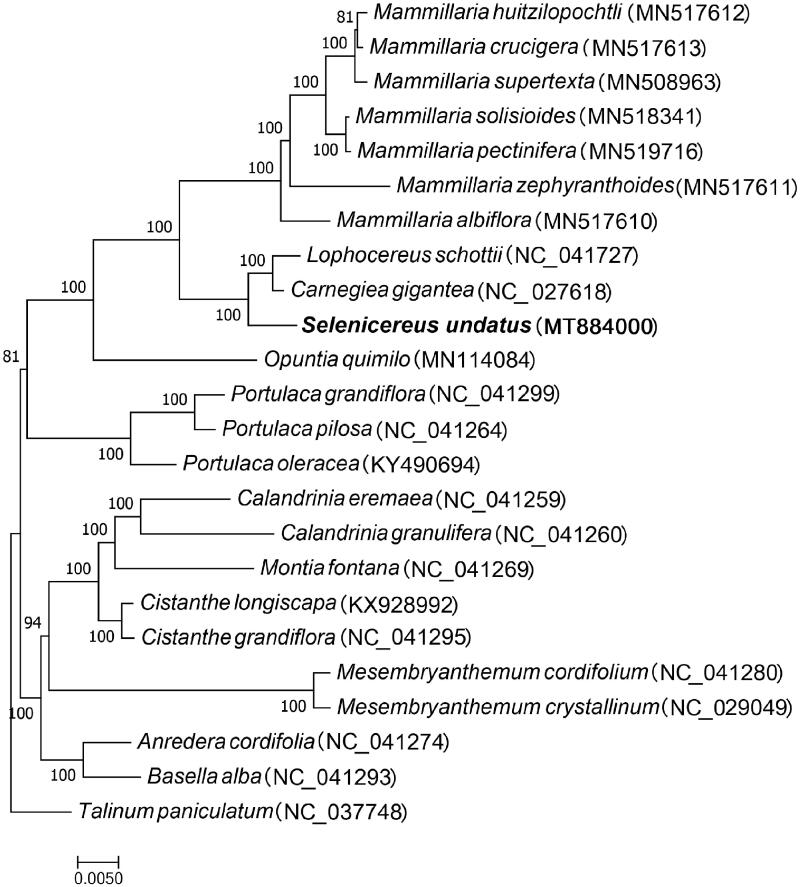
Maximum-likelihood phylogenetic tree of *Selenicereus undatus* and 23 other species, all of the species used to construct the phylogenetic tree belong to the Caryophyllales order, and *Talinum paniculatum*, a species of the Talinaceae family, was used as the outgroup. The bootstrap value was set to 1000.

## Data Availability

The genome sequence data that support the findings of this study are openly available in GenBank of NCBI at (https://www.ncbi.nlm.nih.gov/) under the accession no. MT884001. The associated BioProject, SRA, and Bio-Sample numbers are PRJNA669903, SRR12904121, and SAMN16480738 respectively.
